# A Comparative Analysis of the Electrical Properties of Silicone Rubber Composites with Graphene and Unwashed Magnetite

**DOI:** 10.3390/ma17236006

**Published:** 2024-12-08

**Authors:** Iosif Malaescu, Paula Sfirloaga, Octavian M. Bunoiu, Catalin N. Marin

**Affiliations:** 1 Physics Faculty, West University of Timisoara, Bd. V. Parvan, No. 4, 300223 Timisoara, Romania; iosif.malaescu@e-uvt.ro (I.M.); madalin.bunoiu@e-uvt.ro (O.M.B.); 2 Institute for Advanced Environmental Research, West University of Timisoara (ICAM-WUT), Oituz Str., No. 4, 300086 Timisoara, Romania; 3 National Institute for Research and Development in Electrochemistry and Condensed Matter Timisoara (INCEMC), Dr. A.P. Podeanu No. 144, 300569 Timisoara, Romania; sfirloagapaula@gmail.com

**Keywords:** electrical conductivity, dielectric permittivity, graphene, magnetite, composites, silicone rubber

## Abstract

Three elastomer samples were prepared using GS530SP01K1 silicone rubber (ProChima). The samples included pure silicone rubber (SR), a silicone rubber-graphene composite (SR-GR), and a silicone rubber-magnetite composite (SR-Fe_3_O_4_). The magnetite was synthesized via chemical precipitation but was not washed to remove residual ions. The dielectric response and electrical conductivity of these samples were analyzed across a frequency range of 500 Hz to 2 MHz. The analysis of the complex dielectric permittivity and Cole–Cole plots indicated a mixed dielectric response, combining dipolar behavior and charge carrier hopping. Despite this mixed response, electrical conductivity followed Jonscher’s power law, with the exponent values (0.5 < n < 0.9) confirming the dominance of electron hopping over dipolar behavior in SR-GR and SR-Fe_3_O_4_ samples. The SR-Fe_3_O_4_ sample demonstrated higher dielectric permittivity and electrical conductivity than SR-GR, even though graphene is inherently more conductive than magnetite. This discrepancy is likely due to the presence of residual ions on the magnetite surface from the chemical precipitation process as the magnetite was only decanted and dried without washing. These findings suggest that the ionic residue significantly influences the dielectric and conductive properties of the composite.

## 1. Introduction

Graphene is a two-dimensional material composed of carbon atoms arranged in a hexagonal lattice structure [1]. It is renowned for its exceptional properties, which include remarkable strength, flexibility, and electrical conductivity [2]. Since its discovery in 2004 by physicists Andre Geim and Konstantin Novoselov—who received the Nobel Prize in Physics in 2010 for their pioneering work [3]—graphene has garnered significant interest across various scientific and industrial domains. The applications for graphene are extensive, encompassing fields such as electronics [4], energy storage [5], medical devices [6], and environmental sustainability [7,8]. According to the Market Analysis Report [9], in 2023, the global graphene market was estimated at USD 195.7 million and is expected to increase at a compound annual growth rate (CAGR) of 35.1% from 2024 to 2030.

Graphene has excellent electrical conductivity, making it an ideal material for next-generation electronics [10]. Unlike other conductive materials, graphene’s electrons move quickly and with minimal resistance, allowing for more efficient circuits and faster electronic devices [11]. In contrast to silicon-based electronics that are nearing their physical limits concerning speed and efficiency, graphene presents a viable alternative due to its high electron mobility, which has the potential to facilitate the development of faster and more efficient transistors [12]. Furthermore, graphene’s inherent flexibility and transparency render it particularly suitable for next-generation flexible electronics, including foldable smartphones [13], wearable devices [14], and transparent displays [15]. Additionally, graphene has the capacity to enhance the performance of existing components, such as batteries [16,17], capacitors [18,19], and sensors [20], thereby fostering the creation of more compact and powerful electronic devices.

Magnetite, a naturally occurring iron oxide with the chemical formula Fe_3_O_4_, is distinguished by its unique magnetic properties and conductivity. Characterized by a cubic crystal structure and a distinctive black appearance, magnetite has been the subject of extensive research regarding its potential applications across various fields [21].

One of the most prominent applications of magnetite is its utilization as a magnetic material. Furthermore, its stability and resilience under varying temperature conditions contribute to the durability and reliability of electromagnetic components.

Magnetite is a semiconductor material, but its electrical conductivity can be augmented through various doping processes [22], rendering it suitable for use as an electrode material in solid oxide fuel cells. Additionally, magnetite has been explored as a candidate material for lithium-ion batteries [23]. Its unique structural characteristics facilitate the intercalation of lithium ions, thereby enhancing charge and discharge rates.

Elastomer composite materials represent a promising category of materials for flexible electronics due to their unique combination of properties, including elasticity, durability, and lightweight characteristics [24]. These materials can effectively withstand mechanical stress and deformation while maintaining their structural integrity, making them ideal for applications in wearable technology, flexible displays, and other electronic devices that require adaptability to various shapes and movements.

In contrast to magnetite, which is a cheap material and easy to obtain, a significant challenge associated with graphene pertains to its cost and scalability of obtaining high-quality products [25].

A cheap method of obtaining magnetite is chemical precipitation [26]. If the obtained precipitate is not washed with distilled water and alcohol before drying, traces of salts remain on the surface of the magnetite nanoparticles. The ions of these salts, together with the surface defects, contribute to the increase in the electrical conductivity of the magnetite nanopowder.

This paper presents a comparative analysis of the electrical properties of elastomer composites with graphene and unwashed magnetite in a low-frequency field (from 500 Hz to 2 MHz). The objective of this analysis is to determine whether unwashed magnetite can serve as a substitute for graphene in elastomer composites, without incurring significant changes in permittivity and electrical conductivity, over the investigated frequency range.

## 2. Materials and Methods

Three samples were manufactured for this study: a sample consisting of silicone rubber (denoted by SR), a composite sample consisting of silicone rubber and graphene (denoted by SR-GR), and another composite sample consisting of silicone rubber and magnetite (denoted by SR-Fe_3_O_4_). The silicone rubber is identified by the trade name GS530SP01K1, sourced from ProChima(Prochima, Rome, Italy) [27], and consists of two liquid components (denoted by A and B) that, upon combination, undergo a curing process, solidifying into the desired shape after a duration of 24 h. Graphene nanoplatelet powder was purchased from Sigma-Aldrich and is identified by the code 799084-500MG [28].

The magnetite powder was synthesized by the chemical precipitation of bivalent and trivalent iron salts in an aqueous solution, with an excess of 25% of NH_4_OH. The precipitate was decanted and air-dried on filter paper.

In order to manufacture the SR sample, we mixed 2 g of silicone rubber’s A component with 2 g of silicone rubber’s B component. Then, the mixture was placed in a square parallelepiped mold for 24 h. To obtain the SR-GR sample, we mixed 1.9 g of silicone rubber’s A component, 1.9 g of silicone rubber’s B component, and 0.2 g of graphene (i.e., 5.26 mass percentage of graphene); then, the mixture was placed in a square parallelepiped mold for 24 h. To obtain the SR-Fe_3_O_4_ sample, we mixed 1.9 g of silicone rubber’s A component, 1.9 g of silicone rubber’s B component, and 0.2 g of Fe_3_O_4_ (i.e., 5.26 mass percentage of magnetite). Then, the mixture was placed in a square parallelepiped mold for 24 h. The as-obtained samples had the shape of a square parallelepiped plate with a length of 5 cm and a thickness of 0.1 cm.

The morphology of the samples was investigated with an FEI Inspect S microscope model scanning electron microscopy (SEM). SEM images were analyzed in low vacuum mode, at an accelerating voltage of 25 kV and a spot value of 3, without using a contrast agent.

The real component, ε′, and imaginary component, ε″, of the complex dielectric permittivity, were determined over the frequency range (500 Hz–2 MHz). For this, we used a planar capacitor (with circular plates that were 4 cm in diameter) connected to an LCR meter (Agilent 4980A). The measurements were performed in two stages: with the sample between the plates of the capacitor and with an empty capacitor. The components of the complex dielectric permittivity were computed using the following equations:

(1) $$\varepsilon^{\prime }=\frac{Cp}{Cpo}$$

(2) $$\varepsilon^{\prime \prime }=\frac{1}{2\pi fCpo}\left(\frac{1}{Rp}-\frac{1}{Rpo}\right)$$

where *Cp* and *Rp* are the parallel capacity and the parallel resistance of the capacitor with a sample between the plates, respectively, *Cpo* and *Rpo* are the parallel capacity and the parallel resistance of the capacitor without a sample between the plates, respectively, and *f* is the measurement frequency.

Electrical conductivity, *σ*, was determined from permittivity as:

(3) $$\sigma =2\pi f\varepsilon_{0}\varepsilon^{\prime \prime }$$

where ε_0_ is the dielectric permittivity of free space (ε_0_ = 8.856·10^−12^ F/m). All the measurements were performed at room temperature.

## 3. Results and Discussion

### 3.1. SEM Analysis

**Figure 1:**
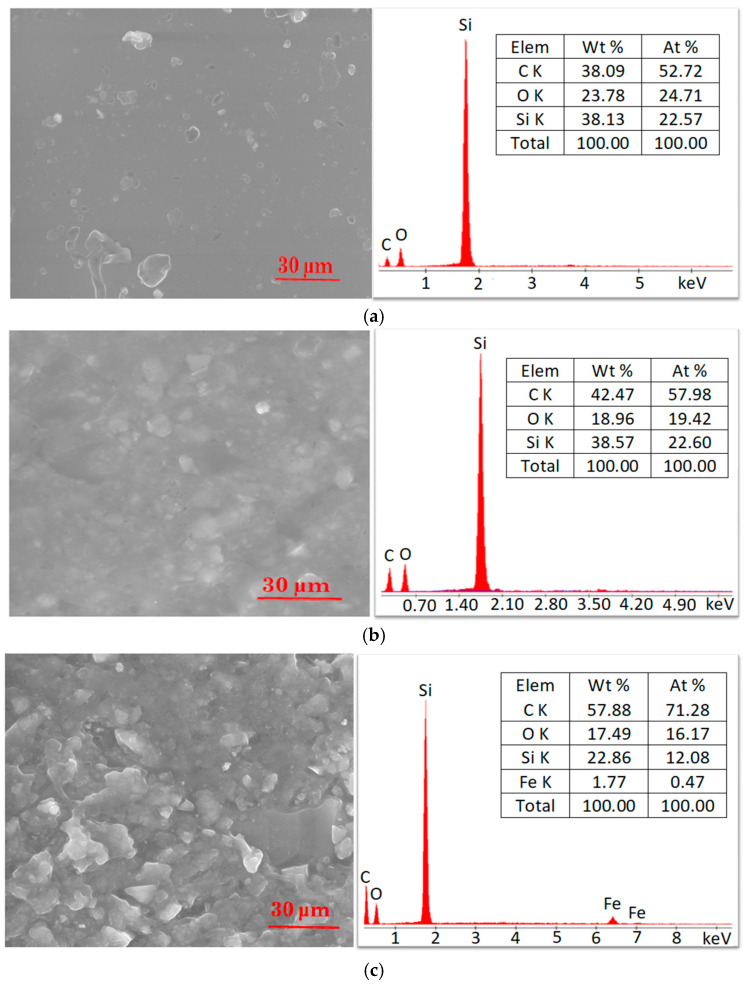
SEM images and elemental analysis of the samples: (**a**) SR sample; (**b**) SR-GR sample; and (**c**) SR-Fe_3_O_4_ sample.

Figure 1 presents scanning electron microscopy images and the spot analysis spectrum, which illustrate the impact of compositional modifications—specifically, the incorporation of graphene and Fe_3_O_4_ in silicone rubber—on the characteristics of samples. In the image related to the silicone rubber sample (Figure 1a), artifacts appear, which give the impression of the presence of some particles, but these are due to the mold, which is not perfectly smooth.

### 3.2. Complex Dielectric Permittivity

**Figure 2:**
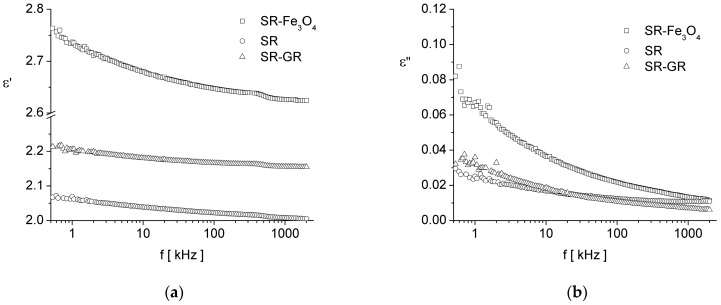
Frequency dependence of the complex dielectric permittivity of samples: (**a**) the real component, ε′(f), and (**b**) the imaginary component, ε″(f).

The results of the frequency dependence of the complex dielectric permittivity of the samples are presented in Figure 2. In this figure, we can observe that in the investigated measurement range, both the real component, ε′, and the imaginary component, ε″, of the complex dielectric permittivity decrease with increasing frequency, *f*.

In broad terms, the materials have two fundamentally distinct types of dielectric responses, leading to the so-called ‘dipolar’ and ‘charge carrier’ behavior [29].

Many experimental results on the frequency dependence of complex dielectric permittivity of materials with dipolar response are discussed in terms of Cole–Cole dispersion equations [30]. According to the Cole–Cole dispersion equations, the imaginary component of the complex dielectric permittivity, ε″, has a maximum, and the real component of the complex dielectric permittivity, *ε′*, decreases from a ε(0) value to a $\varepsilon_{\infty }$ value, having an inflection point at the frequency at which ε″, has a maximum. Sometimes, when dealing with both electrical conduction and dielectric relaxation losses, the term σ/ε_0_ω is added to the imaginary component, ε″, resulting in the frequency dependence of complex dielectric permittivity components, given by Equations (4) and (5) [31]. In Equations (4) and (5), ε(0) is the static dielectric permittivity (measured at frequencies much smaller than the frequency *f_max_*, at which *ε″* has a maximum),$\varepsilon_{\infty }$ is the dielectric permittivity measured at frequencies much higher than *f_max_*, ω=2πf is the pulsation of the measuring electric field, *f* is the frequency of the measuring electric field, τ is the relaxation time (it is characteristic of the dielectric relaxation process), σ is the electrical conductivity of the material, $\varepsilon_{0}$ is the dielectric permittivity of the vacuum, and α is a constant that takes values between 0 and 1.

(4) $$\varepsilon^{\prime }=\varepsilon_{\infty }+\left[\varepsilon (0)-\varepsilon_{\infty }\right]\frac{1+{\left(\omega \tau \right)}^{1-\alpha }\sin\left(\frac{\alpha \pi }{2}\right)}{1+2{\left(\omega \tau \right)}^{1-\alpha }\sin\left(\frac{\alpha \pi }{2}\right)+{\left(\omega \tau \right)}^{2\left(1-\alpha \right)}}$$

(5) $$\varepsilon^{\prime \prime }=\frac{\sigma }{\omega \varepsilon_{0}}+\left[\varepsilon (0)-\varepsilon_{\infty }\right]\frac{{\left(\omega \tau \right)}^{1-\alpha }\cos\left(\frac{\alpha \pi }{2}\right)}{1+2{\left(\omega \tau \right)}^{1-\alpha }\sin\left(\frac{\alpha \pi }{2}\right)+{\left(\omega \tau \right)}^{2\left(1-\alpha \right)}}$$

As shown in Figure 2, the real component, ε′, slowly decreases with frequency, following a power law like ε′ ≈ ω^k−1^, with 0 < k < 1. The dependence ε′(f) has three regions: the first region being below a frequency of approximately 500 kHz, the second region being around 500 kHz, and the third region being above approximately 500 Hz.

We consider that the decrease associated with the frequency of ε′ (below 500 Hz) is due to the movement of charge carriers in the investigated materials by hopping between the localized states, which leads to the gradual accumulation of electrical charges on the surface of the sample [32] (for the SR sample) or at the separation limit between silicone rubber and graphene and between silicone rubber and magnetite particles (for the SR-GR and SR-Fe_3_O_4_ samples, respectively) [32]. The second region, around 500 kHz, exhibits an inflection point, which is characteristic of a dipolar relaxation process, and the one above a frequency of approximately 500 kHz is a region towards the next dielectric relaxation process.

Regarding the imaginary part, ε″, it seems to approach the dependence (5) for a low-frequency regime, ε″ ≈ σ(ε_0_ ω)^−1^, but in fact, the slope of log(ε″) as a function of log(ω) is different from −1. However, as in the case of ε′, the imaginary part of permittivity follows a power law, e.g., ε″ ≈ ω^k−1^, with 0 < k < 1. This type of dependence at low frequencies is characteristic of materials in which electrical conduction by the movement of charge carriers dominates the dielectric response [32].

Similar frequency dependencies of complex dielectric permittivity were reported in Ref [33] for composite samples consisting of RTV silicon rubber, in which fumed silica, with a diameter of a few nanometers, and aluminum trihydrate, with a diameter of a few micrometers, were introduced as fillers.

**Figure 3:**
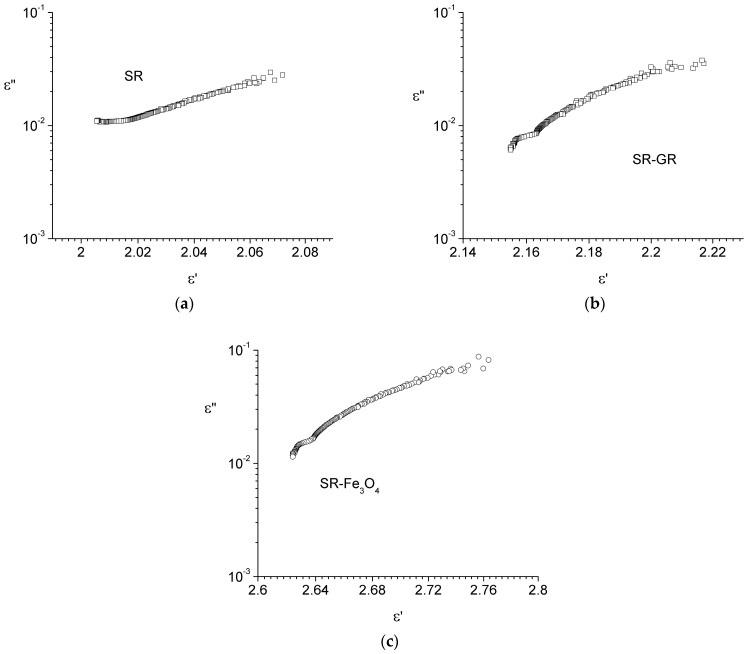
Cole–Cole plots of the samples: silicon rubber (SR)—(**a**), silicon rubber-graphene (SR-GR)—(**b**), and silicon rubber-magnetite (SR-Fe_3_O_4_)—(**c**).

As already mentioned, ε′(f) shows an inflection point around the frequency of 500 kHz, and according to Equations (4) and (5), ε″(f) should exhibit a maximum around this frequency. This maximum is not visible due to electrical conduction losses, which are greater than the relaxation losses and cover the relaxation maximum. In order to support the assertion that a relaxation maximum is covered by the conduction losses around 500 kHz, Cole–Cole dependences are presented in Figure 3. From Figure 3, it can be seen that at low frequencies (below the approximate value of 500 kHz), there is a wide arc, and at high frequencies (around approximately 500 kHz), there is another arc, specific to a relaxation process. In our opinion, the low-frequency arc is due to electrical conduction by hopping of charge carriers [32], and the arc around 500 kHz is due to the relaxation processes in silicone rubber. In Ref [33], it is shown that silicone rubber presents a *β* relaxation peak at frequencies between approximate values 10^4^ and 10^5^ Hz, in agreement with our findings.

Thus, based on the frequency dependencies of complex dielectric permittivity, we can state that over the measuring range, the dielectric behavior is dominated by the charge carriers, or in other words, it is strongly influenced by electrical conduction losses. We can also observe that at low frequencies, up to approximately 3 kHz, the overall dielectric losses (i.e., electrical conduction losses plus dipolar relaxation losses) of the silicone rubber sample are the lowest, and the overall dielectric losses of the composite sample of silicone rubber and magnetite are the highest.

Regarding the values of the real component of the complex dielectric permittivity, it can be observed from Figure 2 that the composite samples have a dielectric constant, ε′, higher than that of silicone rubber. This result is due to the phenomenon of interfacial polarization, also known as Maxwell–Wagner–Sillars polarization [34]. This phenomenon is characteristic of composite materials and is due to the difference between the electrical conductivity of the dispersion medium and the electrical conductivity of the dispersed particles. Thus, the mobile charge carriers accumulate on the surface of the separation surface between conductive particles and the dispersion medium with lower electrical conductivity, which leads to the occurrence of one electric dipole at the physical limit of each conductive particle (or at the physical limit of each agglomeration of particles). Due to the fact that the magnetite nanoparticles were not washed (but only air-dried), traces of ions that did not chemically react in the chemical precipitation process remained on their surface. These ions contribute to the increase in the electric dipole moment of each magnetite nanoparticle, and overall, to the increase in the real component of the complex dielectric permittivity of the composite sample of silicone rubber-magnetite (SR-Fe_3_O_4_).

Thus, even if graphene is a material with higher electrical conductivity than magnetite, the presence of ions (that have not chemically reacted in the chemical precipitation process) on the surface of the magnetite particles leads to higher apparent conductivity of silicone rubber-magnetite (SR-Fe_3_O_4_) than that of silicone rubber-graphene (SR-GR) and higher dielectric permittivity of the SR-Fe_3_O_4_ sample compared to that of SR-GR sample.

### 3.3. Electrical Conductivity

**Figure 4:**
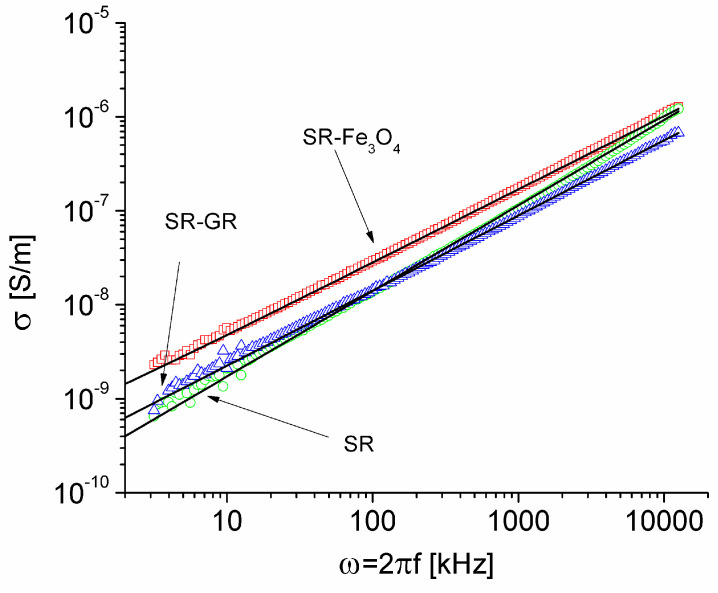
Plots of the electrical conductivity of samples versus ω = 2πf in logarithmic scales and the best fit of Equation (9) to experimental data.

Using the complex dielectric permittivity measurements and Equation (3), the experimental frequency dependence of the electrical conductivity of the three analyzed samples was computed, and the results are presented in Figure 4 (in log–log scales). From Figure 4, one can observe that each dependence is approximately linear. Also, in the approximate frequency range of 200 kHz < f < 2 MHz, the silicone rubber-graphene composite (SR-GR sample) had the lowest electrical conductivity, and the silicone rubber-magnetite composite (SR-Fe3O4 sample) had the highest electrical conductivity.

As shown in Ref. [32], in the case of dipolar dielectric relaxation, the predominant forms of the frequency dependence of permittivity are the fractional power laws:

(6) $$\varepsilon^{\prime \prime }\propto \omega^{m}$$

for the rising part of the relaxation maximum and

(7) $$\varepsilon^{\prime \prime }\propto \omega^{k-1}$$

for the falling part of the relaxation maximum, where *m* and *k* are constants between 0 and 1. In the case of hopping carrier systems, the frequency dependence of the permittivity is as follows:

(8) $$\varepsilon^{\prime \prime }\propto {\left[\ln\left(2\omega_{0}/\omega \right)\right]}^{4}$$

where $\omega_{0}$ is a suitable chosen “vibration frequency” [32,35].

As we discussed in Section 3.2, the dielectric response of the samples is due to both dielectric relaxation and electrical conduction, but the dominant process is that of conduction by hopping of charge carriers. Therefore, σ(ω) represents a superposition of dependencies (originating from Equations like (6), (7), and (8)), arising from the influence of various processes that govern the dielectric response of the samples. In this context, by fitting the Jonscher type dependency (Equation (9)) to experimental data,

(9) $$\sigma \left(\omega \right)=\sigma_{DC}+A\omega^{n}$$

it is possible to determine effective values of the DC conductivity, $\sigma_{DC}$, the pre-exponential factor, A, and the exponent, *n*. These effective values include effects due to conduction as well as dipolar relaxation. The results of the best fit of Equation (9) to experimental data are presented in Figure 4 and Table 1. From Table 1 it can be seen that the effective DC electrical conductivity of silicone rubber is the lowest. The value found experimentally, σ_DC_ = 7.65 × 10^−13^ S/m, is in agreement with the values provided by some silicone rubber manufacturers, who report electrical conductivity values between 10^−14^ S/m and 10^−12^ S/m [36].

At the same time, it can be seen from Table 1 that the addition of graphene to silicone rubber led to an increase in σ_DC_ by 12.3 times, from 7.65 × 10^−13^ S/m to 9.41 × 10^−12^ S/m. Comparable findings were reported by various researchers, demonstrating an increase in electrical conductivity in silicone rubber and graphene composites relative to pure silicone rubber, despite using different types of silicone rubber in their studies [37,38,39,40]. On the other hand, the addition of chemically precipitated and unwashed magnetite to the silicone rubber led to a much greater increase in the static conductivity of the SR-Fe_3_O_4_ composite compared to the silicone rubber (from 7.65 × 10^−13^ S/m to 1.14 × 10^−10^ S/m). In our opinion, the substantial increase in the conductivity of the SR-Fe_3_O_4_ sample compared to that of SR is due both to the electrical conductivity of magnetite [41] and the ions on the surface of magnetite nanoparticles (which remained on the particles after the chemical precipitation process and were not removed by washing). These ions are not mobile in the composite but provide energy states that contribute to the facilitation of the conduction process by electron hopping.

As stated by Jonscher [29,42], values of the exponent, n, between 0.5 and 0.9 are associated with the jump of electrons between localized states in the material, and values of n close to unity are associated with the internal dielectric response of the material by polarization processes. In other words, values of n close to unity are associated with materials that have low electrical conductivity and in which mobile charge carriers play a marginal role.

From Table 1, one can observe that the larger the conductivity, the smaller the exponent, n. The composite samples, SR-GR and SR-Fe_3_O_4_ (in which there are graphene and magnetite with traces of ions), have a dielectric response, which was determined by the hopping of the charge carriers between the localized states in the composite. On the other hand, in the SR sample, which had no conductive impurities, n = 0.91, the dielectric response is less influenced by the hopping of charge carriers.

## 4. Conclusions

Three elastomer samples were prepared based on silicone rubber with the trade name GS530SP01K1 from ProChima. The sample denoted by SR consisted of silicone rubber, the sample denoted by SR-GR was a composite made of silicone rubber and graphene, and the sample denoted by SR-Fe_3_O_4_ was a composite made of silicone rubber and magnetite obtained by chemical precipitation, which was only decanted and dried (without being washed of traces of ions).

For each sample, the dielectric response and electrical conductivity were analyzed, in the frequency range of 500 Hz–2 MHz.

Based on the frequency dependence of the components of complex dielectric permittivity and the analysis of Cole–Cole plots, it can be concluded that the dielectric response of the samples is mixed, consisting of a mixture of “dipolar” and “charge carriers hopping” behavior.

Even if the dielectric response is mixed, the frequency dependence of electrical conductivity obeys Jonscher’s power law. The values of the exponent, 0.5 < n < 0.9, confirm the dominant character of electron hopping in relation to the dipolar one in the case of the SR-GR and SR-Fe_3_O_4_ samples.

The SR-Fe_3_O_4_ sample exhibits greater dielectric permittivity and electrical conductivity compared to the SR-GR sample. While graphene possesses higher conductivity than magnetite, this outcome may be attributed to the presence of residual ions on the surface of the magnetite particles. These ions remained after the chemical precipitation process as the magnetite was not subjected to washing but was merely decanted and dried.

The outcomes of this research could lead to new opportunities in the design of smart materials with enhanced properties for energy storage, flexible electronic devices, or vibration-sensing devices [43]. Also, as mentioned in Ref [44], molecules with magnetic moments that can be modified under an external magnetic field can work as molecular scaffolds to build the next generation of quantum technologies. So, the silicone rubber-magnetite composites with residual ions may be promising materials for the development of new quantum devices. Thermoelectric technology, which can function for both energy generation and rapid cooling [45], is another field of application of silicone rubber-based composites, especially those containing magnetic nanoparticles, which can form local structures under the influence of an external magnetic field [46].

## Figures and Tables

**Table 1:** The effective values of DC conductivity, pre-exponential factor, and exponent of the Jonscher’s power law.

Sample	σ_DC_ [Ω^−1^ m^−1^]	A [Ω^−1^ m^−1^]	n
SR	7.65∙10^−13^	3.95∙10^−13^	0.91
SR-GR	9.41∙10^−12^	1.41∙10^−12^	0.80
SR-Fe_3_O_4_	1.14∙10^−10^	3.53∙10^−12^	0.78

## Data Availability

The raw data supporting the conclusions of this article will be made available by the authors on request.
